# Ignorance is never bliss when it comes to patient care

**DOI:** 10.7196/SARJ.2018.v24i3.226

**Published:** 2018-09-07

**Authors:** Richard van Zyl-Smit

**Affiliations:** University of Cape Town Lung Institute and Division of Pulmonology, Department of Medicine, University of Cape Town and Groote Schuur Hospital, Cape Town, South Africa

## Editorial

Young doctors do not have a lot of experience, and may at times not know what they are doing. This is absolutely understandable and acceptable, as long as they know that they do not know. The doctor who does not appreciate his ignorance is most dangerous. In medical training and education, keeping up to date with new trends in treatment or research can be challenging, especially if it is in an area that one does not frequently encounter. To evaluate the level of knowledge, surveys are often sent out to assess knowledge and practice. Do they know the latest guideline on asthma? Are they treating diabetes according to the latest statement? Frequently, and not unsurprisingly, they are not following current recommendations, as was beautifully illustrated in a slightly dated study performed in the UK in 2007 to assess adherence to the British Thoracic Society (BTS) guidelines for spontaneous pneumothorax. The guidelines, originally published in 1993, were updated in 2003, a pre- and post study was performed after an intensive 60-minute training session outlining the initial audit findings and then doing a re-audit. Somewhat depressingly, the original proportion of guideline deviations (26.9%) was no different to the post-intervention audit (32.1%).^[1]^

In this edition of the *AJTCCM*, Alexander and Perumal^[2]^ report on a survey sent out to pulmonologists on their knowledge and practice regarding the role of adjuvant lung resection for drug-resistant tuberculosis (DR-TB). As could have been expected, the response rate was low, at 50%. The ‘fifty percent response’ elicited 25 forms to evaluate! Therefore the entire list of registered pulmonologists in South Africa (SA) provided to the researchers contained only 50 names. Fifty pulmonologists looking after the lung health of close to 60 million South Africans?

Of the respondents, roughly three-quarters saw less than 10 DR-TB patients a year. The majority of pulmonologists are therefore not managing significant numbers of extensively drug-resistant TB (XDR-TB) patients. Quite surprisingly, the majority still knew the indications for surgery; however, they did not fully appreciate the potential benefits of surgery. The authors have called for a national policy on surgery for DR-TB and a mandatory multi-disciplinary team in all units treating DR-TB.

The suggestion is, unfortunately, unlikely to be feasible as there are only 50 pulmonologists in the country and potentially fewer dedicated thoracic surgeons. This is especially true in the public sector where the majority of these patients are managed. What is needed, however, are clear guidelines for who should be considered for surgery and at what stage of the disease. TB is managed at the primary care level, while multidrug-resistant TB (MDR-TB) and XDR-TB are frequently managed outside of tertiary hospitals. If such guidelines are available, widely disseminated and followed by clinicians (as with the BTS pneumothorax guidelines), management would potentially, but not necessarily, improve.

Surveys always provide problematic data to truly evaluate knowledge and practice, as those who do not know and are not interested are less likely to respond – this inevitably leads to an overestimation of the true level of knowledge. Despite these inherent drawbacks, important information can be obtained, as in the case of this study. These findings should serve as a stimulus to improve our teaching and training across all levels of healthcare. We clearly cannot rely on 50 people to manage the burden of respiratory disease in SA.

MDR-TB occurs in all provinces across SA, where many clinics have no access to surgical or pulmonology expertise. To truly influence the cure rates of DR-TB with appropriate adjuvant resection, education of the indications and basic investigations needs to occur at all levels. Then appropriate referral networks to centres that have the necessary expertise and ability to fully evaluate surgical options with ventilation/ perfusion, and potentially positron emission tomography/computed tomography (PET-CT) scans, need to be developed. In agreement with the authors – with this information at hand – a multidisciplinary team is then empowered to make the appropriate decision on whether to prescribe surgery or not.
